# Immersion Phase Separation 3-Dimensional Printing for Strain-Stiffening Hydrogel Scaffolds

**DOI:** 10.34133/research.0742

**Published:** 2025-06-17

**Authors:** Muyuan Chai, Haolin Bu, Rui Zheng, Zhilu Yang, Xuetao Shi

**Affiliations:** ^1^ Dongguan Key Laboratory of Smart Biomaterials and Regenerative Medicine, The Tenth Affiliated Hospital, Southern Medical University, Dongguan 523059, P. R. China.; ^2^ National Engineering Research Centre for Tissue Restoration and Reconstruction, South China University of Technology, Guangzhou 510006, P. R. China.; ^3^ School of Materials Science and Engineering, South China University of Technology, Guangzhou 510640, P. R. China.; ^4^ Key Laboratory of Biomedical Engineering of Guangdong Province, South China University of Technology, Guangzhou 510006, P. R. China.

## Abstract

Strain-stiffening hydrogels, which mimic the nonlinear mechanical behavior of biological tissues such as skin, arteries, and cartilage, hold transformative potential for biomedical applications. This study introduces immersion phase separation (IPS) 3-dimensional (3D) printing, an innovative technique that enables the one-step fabrication of strain-stiffening hydrogel scaffolds with intricate, hierarchical architectures. This technique addresses the long-standing challenge of balancing structural complexity and intrinsic mechanoresponsive behavior in traditional hydrogel fabrication methods. By leveraging dynamic hydrophobic interactions and solvent exchange kinetics, IPS 3D printing achieves multiscale control over pore architectures (5 to 200 μm) and anisotropic microchannels while preserving J-shaped stress–strain curves (fracture stress: ~0.7 MPa; elongation: >1,000%). The physically cross-linked network enables closed-loop recyclability (>95% material recovery) without performance degradation, while functional fillers (e.g., carbon nanotubes, copper, and hydroxyapatite) enhance properties such as electrical conductivity (2-orders-of-magnitude improvement) and real-time motion sensing capabilities. This platform facilitates the creation of patient-specific implants with tailored mechanical properties and paves the way for adaptive biohybrid devices that mimic the dynamic behavior of native tissues, holding promise for regenerative medicine, soft robotics, and advanced biomedical applications. IPS 3D printing uniquely resolves the trade-off between structural sophistication and functional biomimicry, establishing a paradigm for replicating dynamic biological tissues.

## Introduction

Biological tissues such as skin, arterial walls, and cartilage exhibit strain-stiffening behavior—a nonlinear mechanical response critical for physiological functions under dynamic loading [1]. For example, collagen fibril realignment in the skin prevents overextension, while elastin–collagen networks in arteries stiffen under pulsatile pressure to maintain hemodynamic stability [2–4]. This adaptive stiffening arises from hierarchical microstructures, where molecular-scale interactions (e.g., collagen triple-helix unfolding and proteoglycan sliding) translate to macroscopic mechanical resilience. Natural tissues exhibit hierarchical mechanoresponsiveness, but synthetic hydrogels struggle to replicate this due to inherent trade-offs: structural complexity, scalability, and strain-stiffening behavior often cannot be achieved simultaneously [5–7]. Current strategies, including macroscopic structural engineering or heterogeneous phase integration, often fail to achieve both tissue-like mechanics and customizable 3-dimensional (3D) architectures in a single-step process [8–10].

Our prior research has successfully engineered polyvinyl alcohol (PVA)-based hydrogels functionalized with short alkyl chains (denoted as PVA-C*n*-DS, where C*n* represents the carbon number in the side chains and DS represents the degree of substitution). These hydrogels form stable physically cross-linked networks through solvent/nonsolvent-exchange-induced phase separation [11–14]. Characterized by dynamic hydrophobic associations, these materials exhibit exceptional mechanical robustness (toughness: ~4 kJ/m^2^; fracture stress: ~2 MPa), programmable shape-morphing capabilities, and tissue-mimetic biocompatibility. Notably, they demonstrate intrinsic strain-stiffening behavior analogous to that of biological tissues, a phenomenon attributed to stress-induced alignment of hydrophobic clusters and polymer chain orientation. Through solvent–vapor exchange strategies, we further achieved hierarchical microstructures with tunable pore sizes and anisotropic properties, including optical transmittance exceeding 90%. These advancements highlight their potential for corneal repair, soft robotics, and tissue engineering. However, current fabrication methods (e.g., mold casting or solvent swelling) are limited to simple geometries like films or sheets, which are incompatible with the complex, customized architectures required for 3D printing [15,16]. This critical limitation restricts their application in advanced biomedical fields requiring structural customization, such as patient-specific implants and biohybrid actuators, thereby necessitating innovative manufacturing strategies that integrate geometric complexity with functional biomimicry [17,18].

To date, no 3D printing methodology has demonstrated the capacity for single-step fabrication of hydrogels that simultaneously achieve strain-stiffening behavior and complex architectures [19–21]. This critical gap persists despite recent advancements in phase separation printing techniques such as the diffusion-induced phase separation (DIPS) [22], immersion precipitation 3D printing (ip3DP) [23], polymerization-induced phase separation (PIPS) [24,25], and vapor-induced phase separation (VIPS) [26] methods. Although these approaches enable hierarchical porosity control, they fundamentally compromise either mechanical functionality or structural sophistication. For example, DIPS-derived networks suffer from static noncovalent interactions that limit strain responsiveness [22], ip3DP/VIPS systems produce rigid isotropic structures incompatible with soft tissue mechanics [23,26], and PIPS struggles to balance rapid gelation with viscoelastic performance [24,25]. It is imperative to note that the distinctive hydrophobic interactions present within the PVA-C*n*-DS system, which facilitate strain stiffening through stress-induced cluster alignment, are not compatible with through straightforward adaptation of existing phase separation printing strategies. This necessitates systematic exploration of printing parameters and ink formulations to achieve concurrent structural complexity and biomimetic mechanical adaptability.

## Results and Discussion

### Overview of immersion phase separation 3D printing

**Fig. 1.**
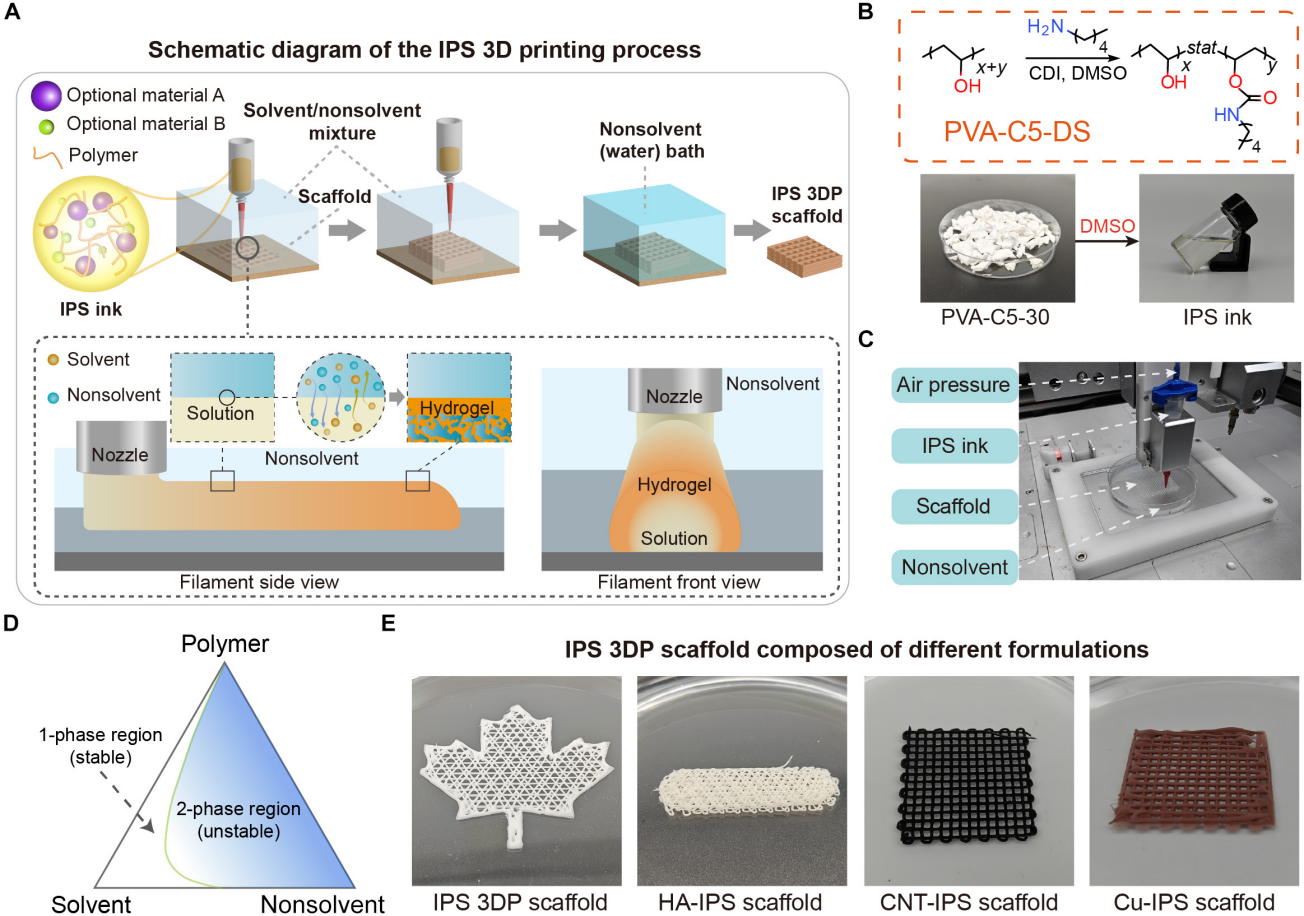
Overview of immersion phase separation 3-dimensional printing (IPS 3DP). (A) Schematic diagram of IPS 3DP, where polymer refers to PVA-C5-DS; solvent refers to dimethyl sulfoxide (DMSO), *N*,*N*-dimethylformamide (DMF), etc.; and nonsolvent refers to water. (B) Synthetic equation of PVA-C5-30, photographs of the PVA-C5-30 samples, and IPS 3DP ink. (C) Representative photographs of the IPS 3DP process. (D) Ternary diagrams for the PVA-C5-30/DMSO/water ternary systems. (E) IPS 3DP scaffolds modified by hydroxyapatite (HA), carbon nanotubes (CNTs), and nano-copper powder doping were used, named HA-IPS scaffold, CNT-IPS scaffold, and Cu-IPS scaffold, respectively. PVA, polyvinyl alcohol; CDI, *N*,*N*′-carbonyldiimidazole.

Leveraging the unique phase separation dynamics of PVA-C*n*-DS, we have developed a novel direct ink writing 3D printing technology called immersion phase separation 3D printing (IPS 3DP). The innovation lies in the strategic modulation of solvent exchange kinetics (i.e., the rate at which the solvent [e.g., dimethyl sulfoxide (DMSO)] in the polymer ink diffuses into the coagulation bath [nonsolvent, e.g., water], driving phase separation and gelation) through the optimization of the coagulation bath composition. This enables precise control over gelation rates and microstructure evolution during extrusion-based printing. Building on our prior work, high-molecular-weight PVA (*M*_w_ 146,000 to 186,000) hydrogels grafted with *n*-pentylamine (C5) side chains were selected as the base material. As shown in Fig. S1, ^1^H nuclear magnetic resonance analysis revealed a side-chain substitution degree of 25% to 35%, and this polymer was named PVA-C5-30. The IPS 3DP ink is prepared by dissolving PVA-C5-30 at specific ratios in anhydrous DMSO (Fig. 1B). As demonstrated in Fig. S2, while IPS 3DP inks can be extruded in air, their slow solvent exchange with water vapor leads to structural collapse during multilayer deposition, precluding compatibility with VIPS-like printing methodologies. Conversely, direct extrusion into aqueous environments induces rapid solvent exchange, causing localized phase separation heterogeneity and viscosity spikes that compromise printing fidelity. Therefore, precise modulation of solvent exchange kinetics and consequent in situ gelation rates—through strategic adjustment of ink concentration, filament diameter, and nozzle translation speed—is paramount for achieving high-fidelity IPS 3DP. For PVA-C5-30, DMSO serves as a good solvent, while water acts as a nonsolvent, with DMSO and water being fully miscible in all proportions. Therefore, we selected DMSO/water mixtures as the coagulation bath for IPS 3DP ink extrusion. By adjusting their ratio, the solvent exchange kinetics can be precisely controlled, enabling high-fidelity IPS 3DP (Fig. 1A). The detailed IPS 3DP process is shown in Fig. 1C and Movie S1. Through systematic analysis of ternary phase diagrams (Fig. 1D), we established printing protocols that decouple shape retention from mechanical performance—overcoming the structural limitations of conventional phase separation methods. Notably, by controlling the diffusion kinetics, IPS 3DP creates hierarchical pore architectures (5 to 200 μm) and anisotropic microchannels. The process also preserves J-shaped stress–strain curves (fracture stress: ~0.7 MPa; elongation: >1,000%), ensuring material integrity and reliability. The physical cross-linking mechanism enables closed-loop recyclability (>95% material recovery) and functional augmentation via inorganic fillers (Fig. 1E). This achieves electrical conductivity enhancements of 2 orders of magnitude for real-time motion sensing applications. Physical cross-linking is defined herein as the reversible formation of dynamic hydrophobic interactions between alkyl side chains. This differs fundamentally from static cross-linking mechanisms, such as hydrogen bonding or ionic interactions. These transient hydrophobic associations give the hydrogel network self-healing capacity and closed-loop recyclability. Moreover, these associations enable strain stiffening through the stress-induced reorganization of cross-linking domains under mechanical loading. It is important to note that the fully physical cross-linking mechanism endows IPS 3DP scaffolds with exceptional recyclability, demonstrating that a closed-loop reprocessing protocol achieves >95% material recovery without performance degradation. Systematic material characterization revealed that the retained strain-stiffening behavior was comparable to that of cast hydrogels.

### IPS 3DP versus slow solvent exchange molding

**Fig. 2.**
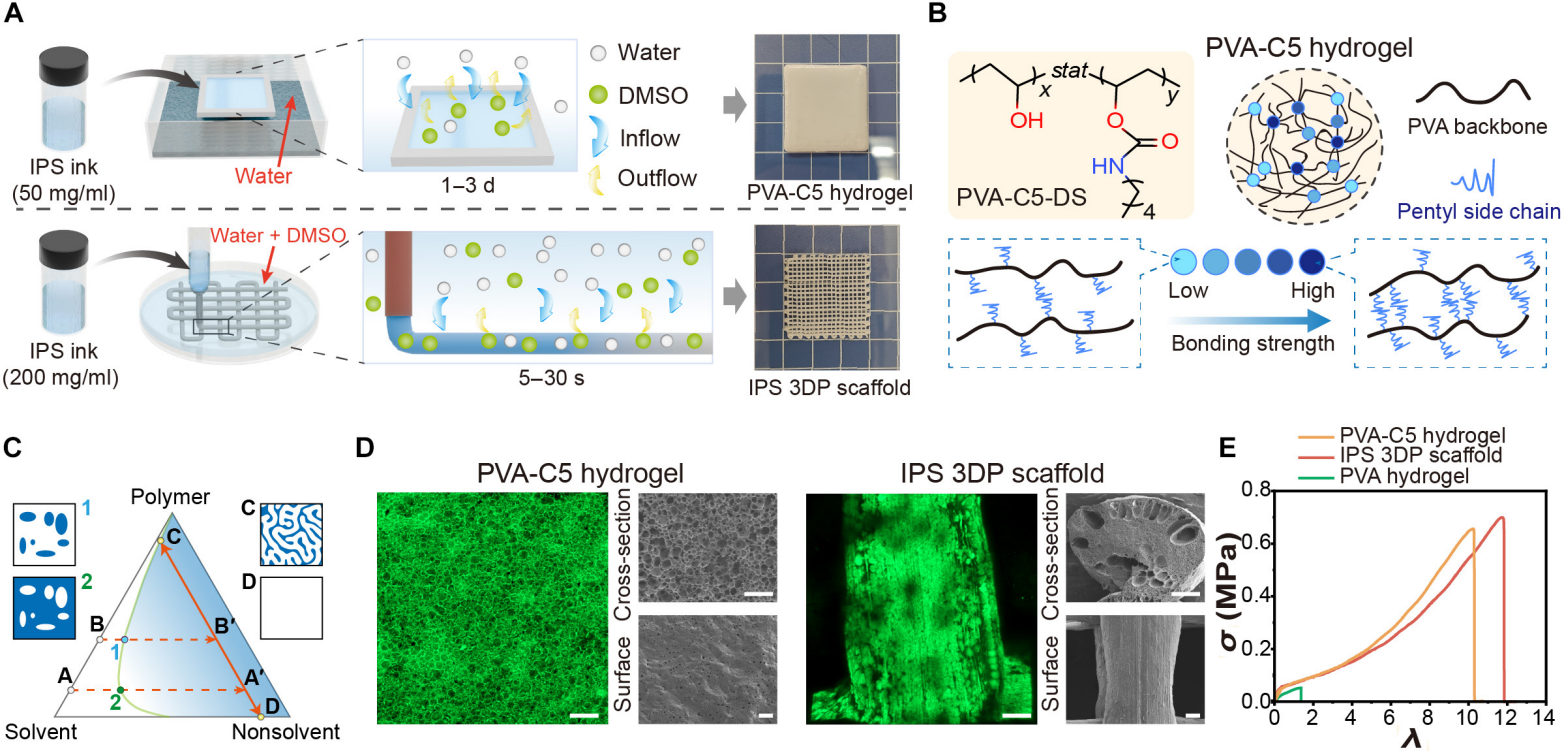
Comparison of the IPS 3DP method with previously reported slow solvent exchange forming methods. (A) Schematic of the process of the 2 forming methods and the final samples obtained, noted as PVA-C5 hydrogel and IPS 3DP scaffold, respectively. (B) Molecular structure of PVA-C5-DS and schematic illustration of hydrogel network formation. (C) Variation paths in the polymer/solvent/nonsolvent ternary phase diagram for the 2 forming methods. (D) Microstructural analysis of PVA-C5 hydrogels and IPS 3DP scaffolds via scanning electron microscopy (SEM) and confocal laser scanning microscopy (scale bar: 100 μm). (E) Stress–strain curves for the uniaxial stretching of a PVA-C5 hydrogel and an IPS 3DP scaffold (a physically cross-linked PVA hydrogel obtained by repeated freeze–thawing was used as the control group).

As schematically illustrated in Fig. 2A, our previous hydrogel fabrication strategy relied on template-based casting, where a 50 mg/ml PVA-C5-DMSO solution was poured into polytetrafluoroethylene (PTFE) molds (≤2-mm thickness) and subjected to slow solvent exchange with saturated water vapor in sealed chambers at 25 °C for 1 to 3 d to induce phase separation, followed by thorough deionized (DI) water immersion to complete cross-linking. This method allows microstructural modulation by controlling solvent exchange rates through humidity adjustment, but the prolonged process fundamentally limits the fabrication of complex 3D architectures, only enabling the production of hydrogel films. To achieve high-fidelity 3D printing of PVA-C5 hydrogels, we developed a strategy to modulate the coagulation bath composition, enabling precisely timed phase separation of the extruded ink within an optimal timeframe. This methodology, termed IPS 3DP, synergizes controlled solvent exchange kinetics with additive manufacturing principles. The ternary phase diagram of the polymer/solvent/nonsolvent (PVA-C5-30/DMSO/water) system (Fig. 2C) reveals distinct gelation pathways for the 2 fabrication methods. The template-cast samples (path A → A′) started from lower polymer concentrations (50 mg/ml) and gradually entered the metastable 2-phase region through controlled vapor absorption. Conversely, IPS inks (path B → B′) utilize higher initial polymer concentrations (200 mg/ml) to maintain extrudability, achieving rapid phase separation upon immersion in aqueous baths. In both cases, solvent depletion drove the system from the single-phase region to the phase-separated state, yielding a continuous porous hydrogel matrix (point C, polymer-rich phase) coexisting with a dilute DMSO/water solution (point D). This fundamental understanding of phase separation guided our design of time-dependent gelation protocols for IPS 3DP, as detailed in the next section.

The microstructural characteristics of the PVA-C5 hydrogels and ice-templated 3D-printed (IPS 3DP) scaffolds were systematically characterized via laser scanning confocal microscopy and scanning electron microscopy (SEM) (Fig. 2D). The PVA-C5 hydrogel exhibited a smooth surface with abundant microchannels formed during slow solvent exchange, whereas cross-sectional analysis revealed a homogeneous porous architecture with pore diameters ranging from 10 to 60 μm. In contrast, the IPS 3DP scaffold displayed hierarchical porosity due to its differential gelation kinetics. The outer layer formed a relatively dense structure through rapid solvent exchange, whereas the intermediate layer was influenced by hindered solvent exchange from the outer shell and outward diffusion of residual DMSO, resulting in larger bubble-like cavities. Importantly, the innermost region of the printed structure remained in a sol state during deposition and achieved complete gelation only via a prolonged postprinting solvent exchange process [26]. This sequential phase separation ultimately resulted in a triphasic architecture featuring a dense exterior, a macroporous intermediate layer, and a microporous core (Fig. S3), suggesting the potential for postfabrication microstructural modulation through controlled solvent exchange protocols—a subject to be further explored in subsequent sections.

Uniaxial tensile testing (Fig. 2E) was used to compare the mechanical behaviors of the PVA-C5 hydrogels and IPS 3DP scaffolds against that of physically cross-linked PVA hydrogels prepared by freeze–thaw cycling. Both the PVA-C5 and IPS 3DP samples demonstrated comparable stress–strain profiles and markedly outperformed conventional physically cross-linked hydrogels (the PVA solution undergoes multiple freeze–dissolve cycle gels) in both elongation at break (1,020% to 1,187%) and fracture strength (0.65 to 0.71 MPa). Notably, after an initial yielding phase, these materials exhibited a characteristic J-shaped stress–strain curve indicative of strain-stiffening behavior. As demonstrated in our previous work [14], this mechanical enhancement originates from the energy dissipation-reinforcement mechanism enabled by gradient-distributed hydrophobic clusters. Short alkyl chains randomly grafted on PVA backbones create dynamic cross-linked networks with a strength hierarchy: weak clusters (low bonding energy) preferentially rupture at low strains to release folded chains for energy dissipation, whereas surviving strong clusters (high bonding energy) progressively align and dominate load bearing at elevated strains, thereby increasing the effective modulus (Fig. 2B).

### Study of IPS 3DP conditions

**Fig. 3.**
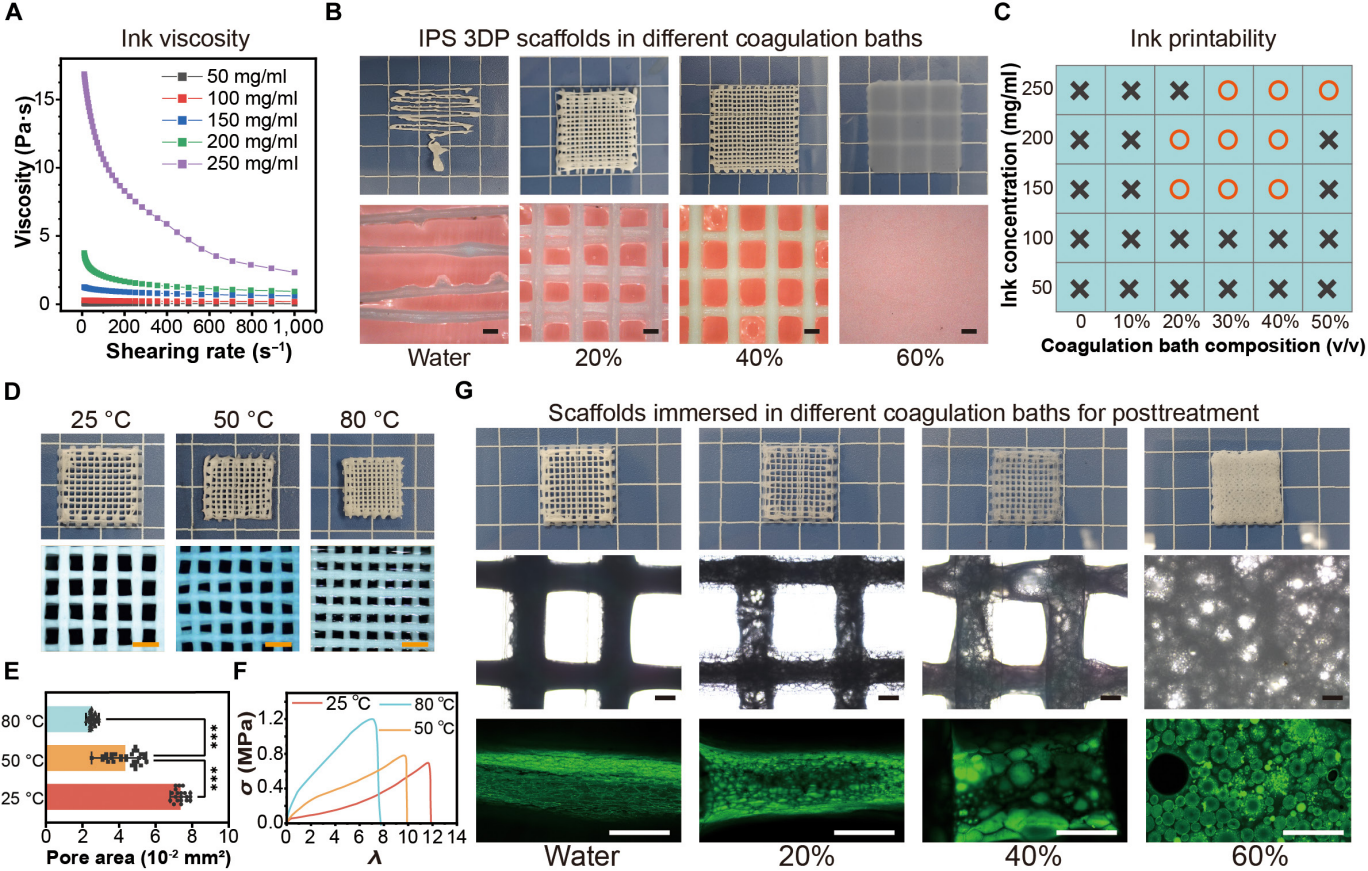
Effects of 3D printing parameters and postprocessing methods on IPS 3DP scaffolds. (A) Rheological characterization of inks with different solid contents. (B) Representative images of IPS 3DP samples prepared with different coagulation bath compositions (water, 20% DMSO, 40% DMSO, and 60% DMSO, v/v). Scale bar: 500 μm. (C) Effect of polymer content in IPS 3DP inks and coagulation bath composition on the printability of the inks (“crosses” in the table mean not printable, and “circles” mean printable). (D) Macro- and microstructural changes in IPS 3DP scaffolds after posttreatment at different temperatures (25, 50, and 80 °C) for 2 h (scale bar: 100 μm). (E) Quantitative analysis of structural dimensional changes in IPS 3DP scaffolds (***P < 0.001). (F) Uniaxial tensile stress–strain curves of posttreated IPS 3DP scaffolds at different temperatures. (G) Macro- and microstructural images of IPS 3DP scaffolds immersed in coagulation baths of various compositions (water and 20%, 40%, and 60% ethanol, v/v) to allow for full solvent exchange following IPS 3D printing (scale bar: 50 μm).

The IPS 3DP ink system has a notably simple composition, comprising solely PVA-C5-30 dissolved in DMSO. We first investigated the rheological dependence on the polymer concentration (Fig. 3A). All formulations demonstrated pronounced shear-thinning behavior with increasing shear rates (0.1 to 100 s^−1^), a critical prerequisite for extrusion-based 3D printing. Quantitative analysis revealed a strong positive correlation between zero-shear viscosity (*η*_0_) and polymer loading: *η*_0_ increased from 0.5 ± 0.1 mPa·s (50 mg/ml) to 15.2 ± 0.8 mPa·s (250 mg/ml) at 10 s^−1^. The 200 mg/ml IPS ink was selected as the standard ink for subsequent experiments because of its superior balance between extrudability and shape retention.

As previously discussed, achieving smooth ink extrusion followed by rapid in situ solidification is critical for high shape fidelity in direct ink writing 3D printing. In IPS 3DP, the phase separation rate is governed by solvent exchange kinetics. Due to the substantially larger mass of the coagulation bath (over 50 times that of the ink) used during IPS 3DP, its composition can be approximately considered invariant. A greater initial DMSO concentration in the ink (approximately 80% for a 200 mg/ml formulation) and a steeper concentration gradient between the ink and coagulation bath may lead to faster solvent exchange rates and earlier phase separation. Notably, DMSO–water solvent exchange is not only influenced by the concentration gradient but also substantially modulated by strong hydrogen bonding interactions between the PVA-C5-30 polymer and DMSO, as well as the dense surface structure formed on the scaffold after gelation. Using a simplified IPS 3DP model, we monitored temporal changes in DMSO concentration within the ink, with results shown in Fig. S4. At varying concentrations, the solvent exchange rates exhibit a distinct 3-stage pattern: (a) an initial rapid decline, (b) steady-state diffusion, and (c) equilibrium approaching. However, for typical IPS 3DP processes (lasting ~2 to 10 min), the focus remains primarily on the first stage. Based on fitted curves, the time required to achieve initial phase separation for self-supporting structures is prolonged by approximately 3-fold and 10-fold in coagulation baths containing 20% and 40% DMSO solutions, respectively, compared to that for pure water.

The precise modulation of the coagulation bath composition enables the precise control of the solvent exchange rate, thereby facilitating the fabrication of high-fidelity scaffolds (Fig. 3B and Movie S2). Pure aqueous baths induce rapid solvent exchange, resulting in inhomogeneous phase separation and poor platform adhesion. Introducing 20% DMSO (v/v) moderated the exchange kinetics, enabling basic lattice formation, albeit with residual defects (~15% strut discontinuity). The 40% DMSO bath achieved ideal gelation dynamics, producing scaffolds with 92.3% ± 2.1% dimensional accuracy versus digital models. Excessive DMSO (60%) suppressed phase separation, delaying gelation beyond the critical time window (>120 s) and causing structural collapse. Combinatorial analysis of the ink concentration and DMSO content (Fig. 3C) revealed a concentration-dependent bath adaptation rule: Lower polymer concentrations (50 to 150 mg/ml) in IPS inks exhibited slower phase separation kinetics, necessitating reduced DMSO concentrations (20% to 30% v/v) in coagulation baths to accelerate solvent exchange rates and ensure appropriate gelation timeframes postextrusion. This empirical relationship provides a universal guide for parameter optimization in solvent-exchange-assisted 3D printing systems.

During IPS 3DP, the solvent exchange kinetics are additionally modulated by the outer shell gel network, which hinders complete solvent exchange throughout the entire scaffold within standard printing timeframes. This inherent kinetic limitation necessitates rigorous postprocessing optimization to regulate scaffold macroscale dimensions, microstructural morphology, and mechanical properties. For example, following IPS 3DP completion, postprocessing at different temperatures markedly modulates the solvent exchange kinetics and the macroscale/microscale structures of the scaffolds, as shown in Fig. 3D. Scaffolds immersed in DI water at elevated temperatures (50 and 80 °C, with 25 °C as control) for 1 h exhibited accelerated solvent exchange kinetics. This rapid phase separation promoted denser polymer network formation—macroscopically manifested as marked volumetric shrinkage—while reducing the water content from 74.76% ± 2.3% (25 °C) to 67.73% ± 1.9% (50 °C) and 61.80% ± 1.6% (80 °C) (Fig. S5). Crucially, this thermal posttreatment not only enhances printing accuracy while maintaining shape fidelity but also imparts improved mechanical properties to the IPS 3DP scaffolds, enhancing dimensional accuracy by 32.4% (Fig. 3E) and elevating the ultimate tensile strength from 0.85 ± 0.12 to 1.43 ± 0.15 MPa (Fig. 3F).

Multiple studies have demonstrated that hierarchical multiscale hydrogels with identical compositions but distinct properties can be fabricated through tunable phase separation [27–30], and this strategy can also be leveraged in IPS 3DP to achieve comparable effects. Postprinting immersion protocols were systematically investigated through 6-h treatments in ethanol/water coagulation baths (Fig. 3G). Macroscopically, the IPS 3DP scaffolds maintained structural integrity except for the 60% ethanol group, where marked collapse occurred due to hindered DMSO diffusion from the core region disrupting the established surface cross-links. Confocal laser microscopy analysis revealed a positive correlation between porosity and ethanol concentration in the coagulation baths (Fig. S6): aqueous-treated scaffolds exhibited dense matrices (porosity 17.3% ± 3.4%), while 40% v/v ethanol baths induced hierarchical pore architectures (porosity 68.7% ± 7.4%, pore size 50 to 200 μm). This solvent–gradient strategy enables precise pore engineering while preserving global geometry—a critical advancement for tissue engineering applications requiring spatially controlled permeability.

### Inorganic-filler-modified IPS 3DP ink

**Fig. 4.**
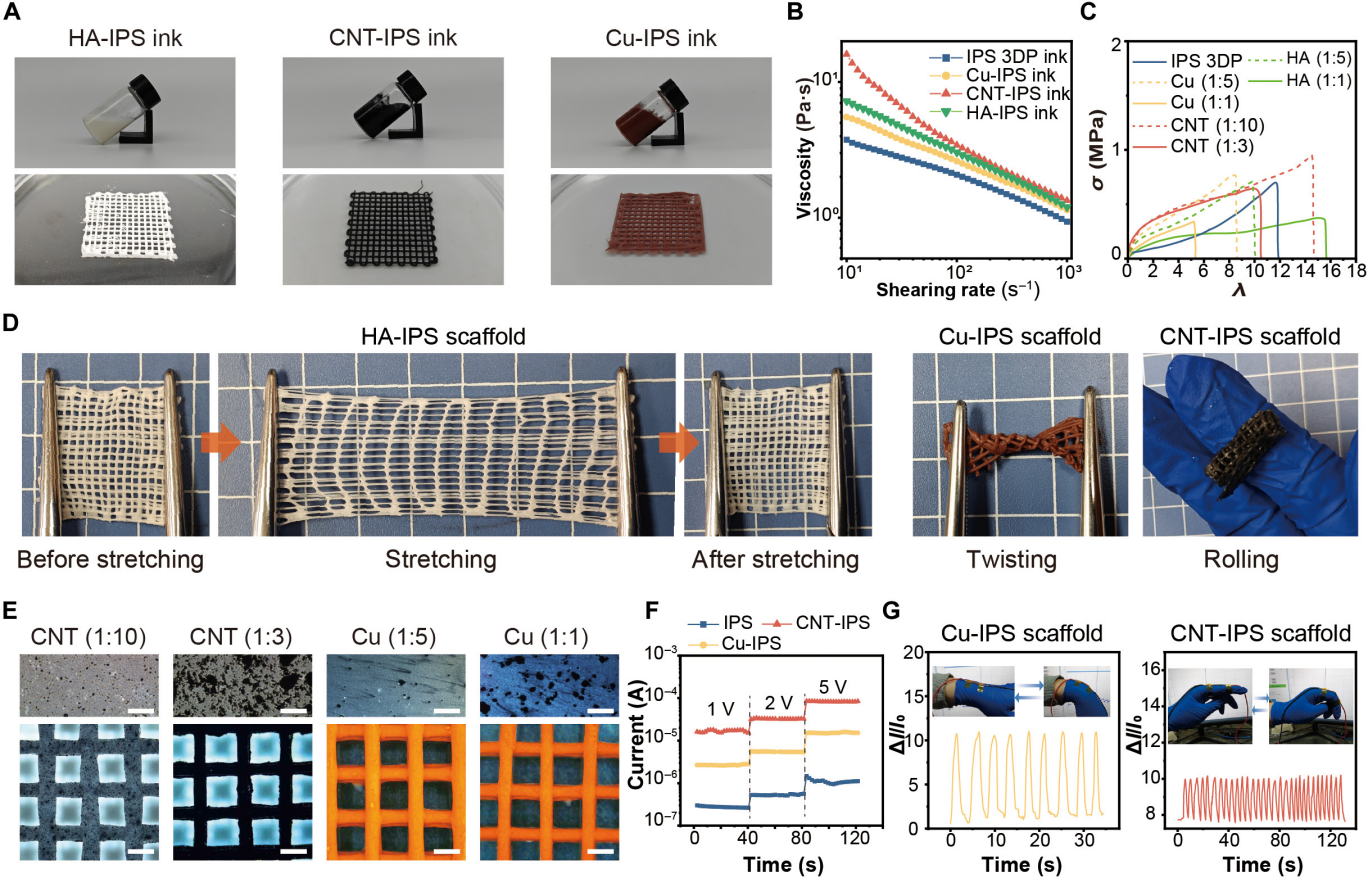
Printing and scaffold characterization of inorganic-filler-modified IPS 3DP inks. (A) Photographs of inks incorporated with hydroxyapatite, carbon nanotubes, and copper powder alongside printed scaffolds. (B) Rheological characterization of inks with various doping modifications. (C) Uniaxial tensile stress–strain curves of IPS 3DP scaffolds fabricated from different composite inks. (D) Stretching, twisting, and curling of different IPS 3DP scaffolds. The white grid in the background consists of squares with a 10-mm side length. (E) Microscopic images of CNT-IPS and Cu-IPS inks and the corresponding printed scaffolds at different doping ratios (scale bar: 100 μm). (F) Effect of different inorganic filler doping modifications on the electrical conductivity of IPS 3DP scaffolds. (G) The Cu-IPS scaffold and CNT-IPS scaffold are used as wearable sensors to monitor hand and wrist movements.

The bioinert properties of PVA-C5-30 may limit the application scope of IPS 3DP scaffolds. However, the IPS 3DP ink system serves as a versatile platform for functional augmentation through doping with one or multiple inorganic fillers (e.g., hydroxyapatite [HA], carbon nanotubes [CNTs], or copper powder), endowing the scaffolds with distinct functionalities tailored to diverse biomedical applications such as osteogenic induction, electrical conductivity enhancement, or dynamic sensing capabilities [26,31–33]. HA (particle size: 200 nm), copper powder (200 to 300 nm), and CNTs (outer diameter 60 to 100 nm, length 5 to 15 μm) were introduced as representative inorganic fillers into the polymer matrix (Fig. 4). All nanoscale fillers formed stable composite inks, designated HA-IPS, CNT-IPS, and Cu-IPS inks, which retained printability in the coagulation bath when the standard IPS 3DP protocol was used (Fig. 4A and Movie S3). Rheological characterization revealed that all of the modified inks maintained a pronounced shear-thinning behavior comparable to that of the unmodified IPS 3DP ink, albeit with elevated viscosities (Fig. 4B). Notably, the viscosity of the CNT-IPS ink increased the most dramatically, which was likely attributable to extensive hydrogen bonding between the CNTs and PVA chains [34,35].

The mechanical effects of filler loading ratios were systematically investigated through 2 distinct formulations: high-loading (HA/polymer = 1:1; Cu/polymer = 1:1; CNT/polymer = 1:3) and low-loading systems (HA/polymer = 1:5; Cu/polymer = 1:5; CNT/polymer = 1:10). Uniaxial tensile testing revealed that low-loading scaffolds maintained the characteristic J-shaped stress–strain curves of the original IPS 3DP scaffolds while demonstrating increased fracture strength (Fig. 4C), suggesting preserved polymer network integrity. In contrast, high-loading scaffolds exhibited reduced fracture strength and diminished strain-stiffening behavior, indicative of mechanical degradation caused by excessive filler content. This deterioration likely originates from the inhomogeneous dispersion of inorganic particles within the high-viscosity ink, resulting in structural defects, a hypothesis supported by optical microscopy observations (Fig. 4E). Microscopic analysis revealed a uniform filler distribution with minimal agglomeration in the low-loading CNT-IPS and Cu-IPS formulations compared with pronounced particle clustering in their high-loading counterparts. These aggregates not only impair mechanical integrity but also increase susceptibility to nozzle clogging during printing. While advanced processing techniques (e.g., ball milling) might address dispersion challenges, such approaches remain beyond the scope of current experimental methods. Notably, despite these microstructural limitations, high-loading composites maintained macroscopic mechanical functionality, demonstrating flexibility through stretching, twisting, and rolling motions (Fig. 4D), confirming the broad application potential of the IPS 3DP system.

While PVA-C5-DSs exhibit bioinert properties, their limited functionality can be augmented through inorganic filler integration. The incorporated additives serve as functional components, endowing 3D-printed scaffolds with tailored properties such as enhanced electrical conductivity[36,37]. As illustrated in Fig. 4F, electrical conductivity measurements revealed distinct performance differences among IPS 3DP scaffolds (9.1 × 10^−4^ ± 2.1 × 10^−4^ S/m), CNT (1:3)-IPS scaffolds (6.64 × 10^−2^ ± 3.5 × 10^−3^ S/m), and Cu (1:1)-IPS scaffolds (1.06 × 10^−2^ ± 0.4 × 10^−3^ S/m) under a 1- to 5-V bias. These results confirm that the incorporation of CNTs and Cu drastically improved the conductivity by 2 orders of magnitude. Further exploration demonstrated the sensing capabilities of the CNT (1:3)-IPS and Cu (1:1)-IPS scaffolds (Fig. 4G). The modified scaffolds exhibited sensitive real-time responses to wrist and finger flexion movements, highlighting their potential for wearable health monitoring or implantable medical devices requiring dynamic biomechanical sensing.

### Recycling and regeneration of IPS scaffolds

**Fig. 5.**
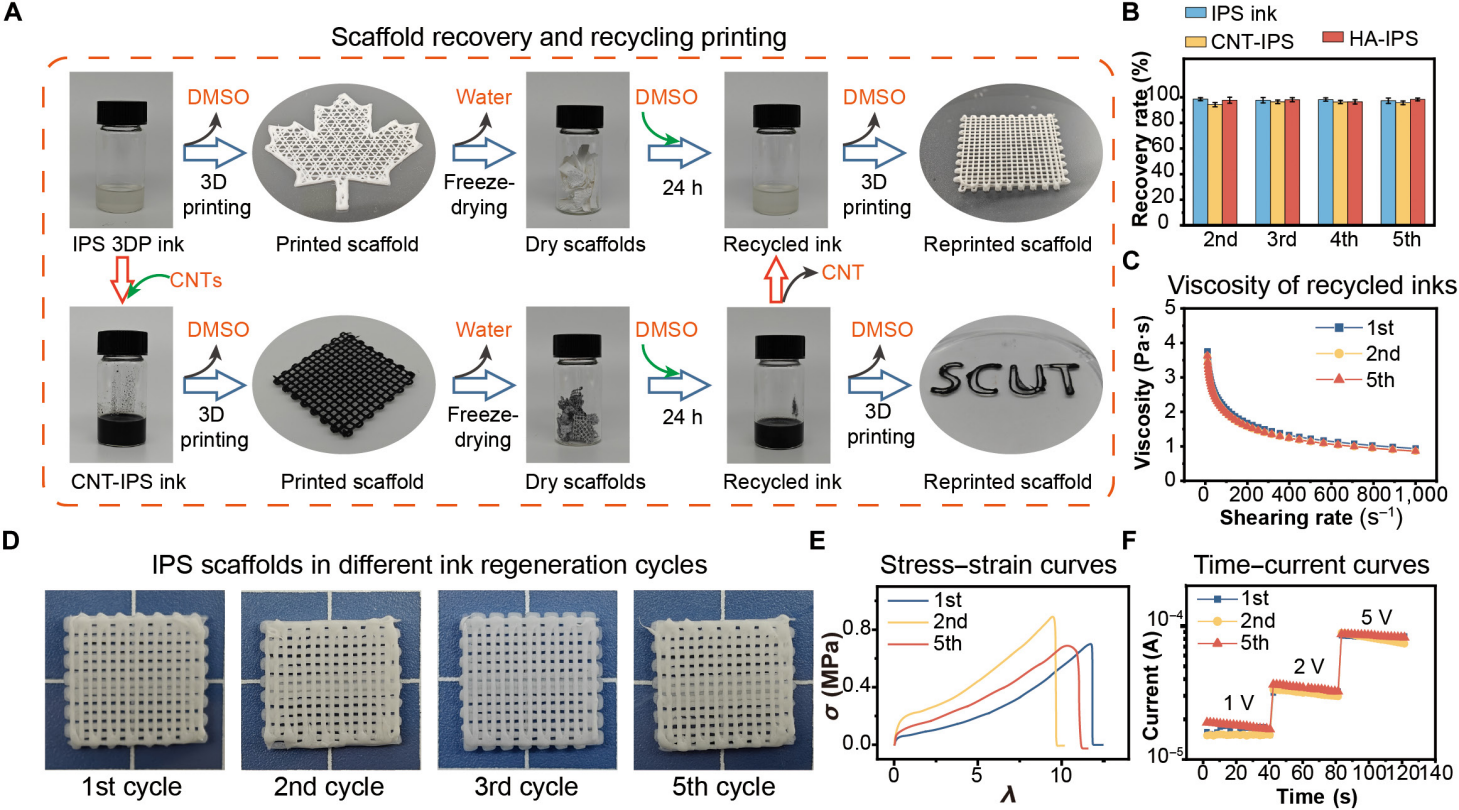
Recyclability and the recycling–ink regeneration–3D printing cycles of IPS 3DP scaffolds. (A) Closed-loop recycling process of IPS 3DP ink and CNT-IPS ink: 3D printing–recycling–ink regeneration–reprinting. (B) Recovery rates (in terms of polymer mass) of IPS 3DP ink, CNT-IPS ink, and HA-IPS ink over 5 ink regeneration cycles. (C) Viscosity changes in IPS 3DP ink over 5 ink regeneration cycles. (D) Appearance of IPS 3DP scaffolds in 5 ink regeneration cycles. (E) Uniaxial tensile stress–strain curves of the initial IPS 3DP scaffold and the reprinted IPS 3DP scaffold. (F) Conductivity of the CNT-IPS scaffolds over different reprinting cycles.

In conventional hydrogel 3D printing, widely used ink systems (e.g., gelatin methacryloyl, hyaluronic acid methacrylate, and polyethylene glycol diacrylate) predominantly rely on irreversible covalent cross-linking [38]. This inherent limitation inevitably leads to complete structural failure and material waste when printing defects occur during layer-by-layer deposition. Although physically cross-linked hydrogels with reversible bonds (e.g., hydrogen bonds or ionic interactions) demonstrate recyclability to circumvent this issue, their compromised mechanical strength severely restricts practical applications, particularly in load-bearing scenarios [22,39]. We present an innovative IPS 3DP ink system that strategically leverages hydrophobic interactions to form physical cross-linking networks, achieving an optimal balance between mechanical robustness and closed-loop recyclability. This design effectively addresses sustainability challenges in additive manufacturing. As systematically validated in Fig. 5A and Fig. S7, the recycling protocol involves 3 streamlined steps: (a) freeze-drying printed scaffolds at −80 °C for 24 h to remove residual DMSO; (b) homogenizing lyophilized scaffolds with DMSO at a 1:5 mass ratio, followed by 24-h dissolution at room temperature to regenerate printable ink; and (c) reprinting the regenerated ink under identical conditions to fabricate recycled scaffolds. Notably, CNT-IPS inks can directly undergo the same printing–regeneration–reprinting cycle. Alternatively, CNT fillers can be removed via centrifugation and filtration during ink regeneration, yielding pristine IPS 3DP ink for reuse.

The recyclability of regenerated IPS inks and 3D-printed scaffolds was systematically evaluated through 5 consecutive printing–recycling–regeneration–reprinting cycles (designated the 1st to 5th cycles, with virgin IPS ink serving as the initial reference). Notably, the Cu-IPS ink was excluded from cycling tests because its stable cross-linked network formation fundamentally impedes regeneration. Recovery rates for IPS, CNT-IPS, and HA-IPS inks were calculated on the basis of mass ratios between recycled scaffold dry weights and original polymer/filler components (Fig. 5B). All systems exhibited exceptional retention values exceeding 95% across cycles, with observed mass losses (<5%) primarily attributable to residual material retention in printing cartridges and nozzles during extrusion processes. Rheological characterization revealed preserved shear-thinning behavior of the cycled IPS inks (Fig. 5C), with negligible viscosity variations observed between the 1st, 2nd, and 5th cycle samples (*P* > 0.05). Macroscopic evaluation of the orthogonal grid scaffolds (Fig. 5D) confirmed that shape fidelity was maintained throughout 5 regeneration cycles under identical printing parameters. Crucially, the mechanical integrity (Fig. 5E) and electrical conductivity (Fig. 5F) of the recycled scaffolds were not notably lower than those of the initial samples. These results collectively validate the robust recyclability of IPS 3DP systems, effectively mitigating material waste from high-cost functional inks while maintaining critical performance metrics.

### Biological research on IPS scaffolds

Since IPS 3DP enables underwater printing that mimics physiological wet environments, we simulated direct tissue printing through in vitro experiments using porcine skin substrates. By employing an ink formulation containing 150 mg/ml polymer and 30 mg/ml CNTs with 20% DMSO as the coagulation bath and porcine skin mounted on plastic culture dishes, we successfully achieved direct on-tissue deposition (Fig. S8). However, practical implementation revealed suboptimal scaffold–tissue adhesion due to lipid interference, suggesting that in vivo applications would require supplemental fixation methods such as sutures or bioadhesives.

**Fig. 6.**
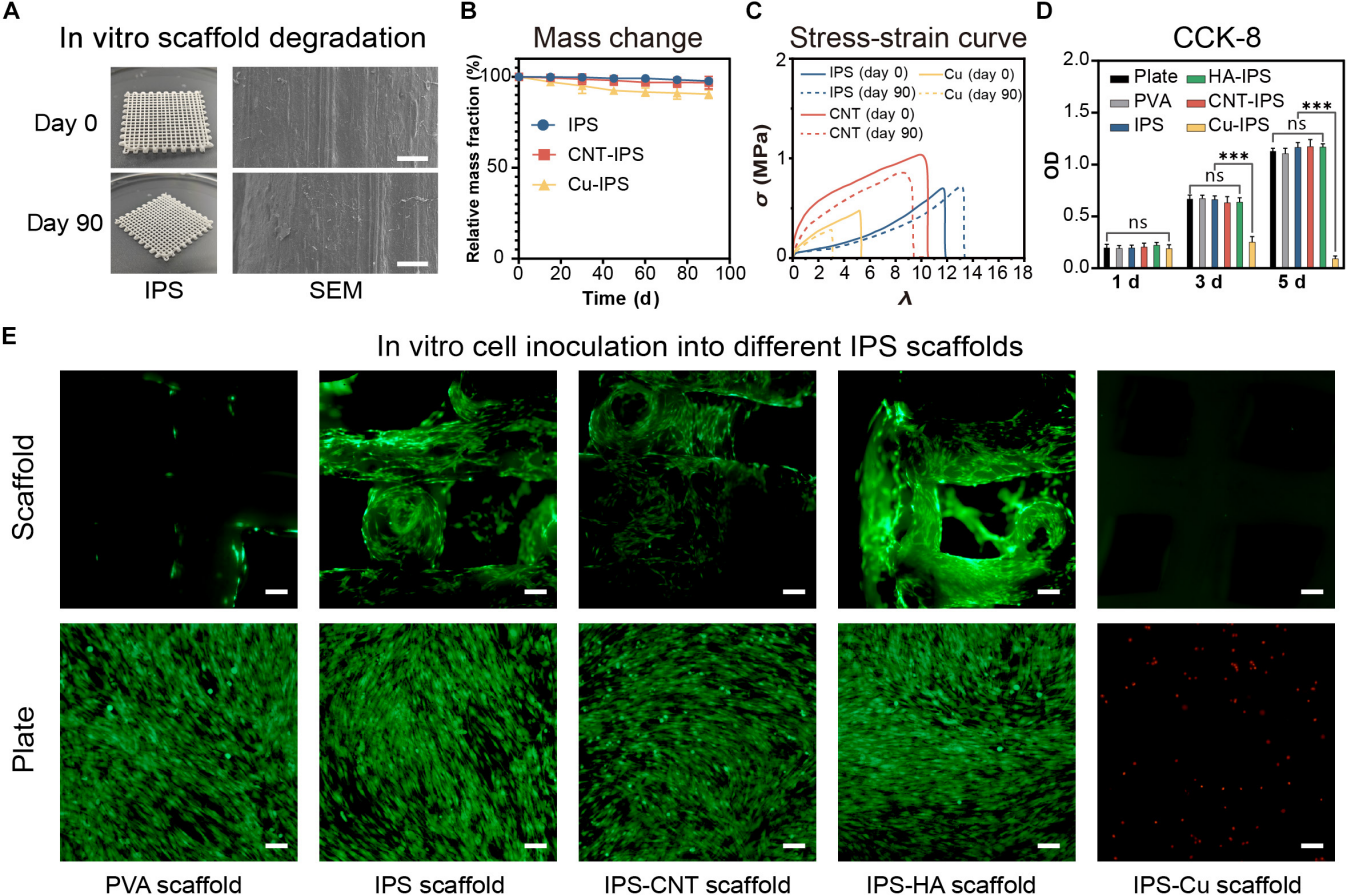
In vitro biological evaluation of IPS 3DP scaffolds. (A) In vitro degradation profiles of IPS 3DP scaffolds, including macroscopic images and SEM micrographs at 0 and 90 d (scale bar: 50 μm). (B) Mass change (dry weight, *n* = 3) of IPS 3DP scaffolds, CNT-IPS scaffolds, and Cu-IPS scaffolds during 90 d in vitro degradation. (C) Comparison of the mechanical properties of IPS 3DP scaffolds, CNT-IPS scaffolds, and Cu-IPS scaffolds before and after 90 d of degradation. (D) Cytocompatibility assessment via a Cell Counting Kit-8 (CCK-8) assay with L-929 fibroblasts (ns, *P* ≥ 0.05; ***P < 0.001). (E) Live/dead staining of bone marrow mesenchymal stem cells (BMSCs) directly seeded onto various scaffolds (intrascaffolds) and adjacent culture plate regions (extrascaffolds). Scale bar: 100 μm. OD, optical density.

In our prior work, we systematically evaluated the in vitro and in vivo long-term biocompatibility of PVA-C*n*-DS materials [1,15]. The results confirmed their nature as stable bioinert materials, demonstrating sustained mechanical stability in physiological environments without substantial biological toxicity or immune rejection. For IPS 3DP scaffolds, degradation studies comparing pure IPS, CNT-IPS, and Cu-IPS scaffolds under simulated physiological conditions (37 °C, enzymatic environment) revealed exceptional stability. After 90 d of in vitro degradation, the macroscopic morphology and microstructure remained unchanged (Fig. 6A and B and Fig. S9), with all scaffolds maintaining structural integrity and exhibiting negligible mass loss (Fig. 6C). These results align with our previous findings on the biostability of IPS scaffolds.

While the nondegradable nature of IPS 3DP scaffolds limits their application in transient tissue regeneration, their long-term mechanical stability makes them ideal for bioelectronic sensors or as functional replacements for load-bearing tissues (e.g., cornea, tunica albuginea, and blood vessels), where durable mechanical support is critical. The scaffolds’ resistance to degradation stems from 2 key features: (a) the inherent chemical inertness of the PVA-C5 backbone, which, structurally analogous to polyethylene with hydrophilic hydroxyl groups, lacks enzymatic cleavage sites for common proteases (e.g., collagenase and hyaluronidase) and (b) the noncovalent dynamic physical cross-linking via transient hydrophobic interactions of *n*-pentyl side chains. Unlike hydrolyzable covalent networks, these entropy-driven molecular associations are not susceptible to enzymatic attack. This structural design explains the negligible mass loss (<5%) and preserved mechanical properties over 90 d (Fig. 6A to C). While advantageous for long-term applications, the nondegradability necessitates future modifications—such as introducing enzymatically responsive moieties (e.g., ester linkages) into the PVA backbone or side chains—to enable tunable degradation kinetics while retaining the strain-stiffening behavior and printability of the IPS 3DP platform.

Given the complex composition of IPS 3DP inks containing residual organic solvents and inorganic fillers, comprehensive in vitro biocompatibility assessments were conducted. Bone marrow mesenchymal stem cells (BMSCs) were selected for Cell Counting Kit-8 proliferation assays to evaluate cellular responses to scaffold extracts in complete culture medium. As shown in Fig. 6D, the experimental groups (IPS, CNT-IPS, and HA-IPS scaffolds) exhibited growth patterns comparable to those of the control groups (blank wells and noncytotoxic PVA hydrogels), indicating minimal cytotoxicity. The observed growth inhibition in Cu-IPS groups likely resulted from copper ion leaching, a hypothesis corroborated by live/dead staining revealing predominantly dead cell populations in Cu-IPS cultures (Fig. S10). Direct cell–scaffold interaction studies using L-929 fibroblasts further validated these findings (Fig. 6E). Despite the inherent biocompatibility of the hyperhydrophilic PVA hydrogel matrix, its lack of adhesion motifs prevented cellular attachment. In contrast, IPS, CNT-IPS, and HA-IPS scaffolds supported robust cell adhesion, attributed to hydrophobic interactions between PVA-C5-30 side chains and cellular components. The complete absence of viable cells on Cu-IPS surfaces, coupled with pronounced peripheral apoptosis, reinforced our conclusions regarding the cytotoxicity of copper ions. This phenomenon is attributed to Cu^2+^ ion leaching, a well-documented limitation of copper-based materials in biological systems. In the context of biomedical applications, it is important to note that, while copper doping demonstrates potential for electrical conductivity, its cytotoxicity underscores the necessity of carefully selecting functional fillers for specific applications. As a modular platform, IPS 3DP allows flexible substitution of fillers. Copper can be replaced with biocompatible alternatives, such as CNTs (at low loading ratios), bioactive glass, or graphene oxide, that can provide similar properties (e.g., conductivity and mechanical reinforcement) without compromising safety. This adaptability highlights the technology’s potential to balance functionality and biocompatibility for various biomedical applications, ranging from wearable sensors to tissue engineering constructs. While CNTs are reported to exhibit potential cytotoxicity [40], the stable hydrogen bonding between CNTs and PVA-C5-30 in our system likely minimized nonspecific cellular interactions, thereby reducing cytotoxic effects. These results underscore the necessity for cautious selection of functional fillers in future biomedical applications of IPS 3DP scaffolds, with careful consideration of their bio-toxicological profiles.

## Conclusion

This study proposes an IPS 3DP strategy for fabricating strain-stiffening hydrogel scaffolds with biomimetic mechanical adaptability. This innovative methodology resolves conventional limitations in reconciling structural complexity with mechanical performance, enabling the creation of hierarchical architectures (pore sizes ranging from 5 to 200 μm; anisotropic microchannel networks) while maintaining the J-shaped stress–strain behavior characteristics of biological tissues, with fracture strengths of 0.65 to 0.71 MPa and elongations at break of 1,020% to 1,187%. The fully physically cross-linked network facilitates closed-loop recyclability (>95% material recovery through 5 reprocessing cycles) and functional versatility through the integration of inorganic fillers (e.g., CNTs for enhanced electrical conductivity and HA for osteogenic enhancement). In personalized biomedical implants, IPS 3DP holds promise for fabricating patient-specific constructs such as cartilage repair scaffolds, vascular grafts, and bone implants. For example, its strain-stiffening behavior mimics the mechanical responsiveness of native cartilage, while its hierarchical porosity (5 to 200 μm) promotes cell infiltration and extracellular matrix deposition for tissue regeneration. The ability to incorporate bioactive fillers like HA can further enhance osteoconductivity for bone defect repair. However, challenges such as long-term in vivo stability, interfacial integration with host tissues, and scalable production of anatomically complex geometries require further optimization. In adaptive soft robotics, IPS 3DP enables the development of biomimetic actuators and sensors. For instance, CNT-doped scaffolds with enhanced electrical conductivity can serve as self-sensing soft grippers, dynamically adapting to object shapes under mechanical stress while transmitting real-time strain data. The material’s high elongation (>1,000%) and programmable anisotropy also facilitate the design of deformable robots for minimally invasive surgery or environmental exploration. Key challenges include integrating IPS 3DP structures with compatible actuation systems, improving mechanical durability under cyclic loading, and achieving multimaterial functionalization for complex robotic tasks.

These advancements establish IPS 3DP as a transformative platform for personalized biomedical implants and adaptive soft robotics. Several limitations of the current study warrant consideration. Firstly, the extrusion-based IPS 3DP process is constrained by nozzle dimensions and ink rheology, limiting the resolution of microscale features. Secondly, the existing coagulation bath system demonstrates poor compatibility with overhanging structures, restricting geometric freedom for 3D-printed models. Thirdly, the incorporation of functional fillers may adversely affect the biocompatibility or mechanical performance of IPS 3DP scaffolds, necessitating careful selection of fillers tailored to specific biomedical applications. Future work will focus on refining multiscale nozzle designs and adaptive path-planning algorithms to enhance structural resolution while exploring hybrid bath compositions to enable support-free printing of complex geometries or thin-walled structures. By bridging these gaps through multidisciplinary innovation, IPS 3DP could unlock new possibilities for patient-specific regenerative medicine and biohybrid device engineering.

## Materials and Methods

### Materials

PVA (*M*_w_ 146,000 to 186,000, 99%+ hydrolyzed) and 1,1′-carbonyldiimidazole (≥90.0%, for peptide synthesis) were purchased from Sigma-Aldrich (Shanghai, China). DMSO (99.7%, with molecular sieves, water ≤50 ppm), amylamine (AR, 98%), nano-HA (particle size: 200 nm), copper (99.9% metal basis, 200 to 300 nm), and CNTs (compound walled, ≥95%, outer diameter 60 to 100 nm, length 5 to 15 μm) were purchased from Shanghai Macklin Biochemical Co., Ltd. (Shanghai, China). Cell Counting Kit-8 and calcein AM/propidium iodide double-staining kits were purchased from Beijing Solarbio Technology Co., Ltd. (Beijing, China). L-929 mouse fibroblasts were purchased from Wuhan Pricella Biotechnology Co, Ltd. (Wuhan, China). All reagents were used without further purification.

### Synthesis of PVA-C5-DS

PVA-C5-DS was synthesized via a one-pot multistep method, as described in our previous work [14]. PVA (5 g, 0.1135 mol of hydroxyl groups) was first dissolved in 100 ml of DMSO at 90 °C for 2 h, followed by natural cooling to ambient temperature. Under controlled thermostatic conditions (20 to 24 °C), 6.5 g of 1,1′-carbonyldiimidazole was added incrementally with continuous agitation for 1.5 h. Subsequently, 7 ml of *n*-pentylamine (diluted in 40 ml of DMSO to prevent localized concentration gradients) was introduced via a pressure-equalizing dropping funnel for 30 min, and the substitution reaction proceeded for 24 h under an inert atmosphere, with the substitution efficiency monitored by ^1^H nuclear magnetic resonance spectroscopy. The reaction was terminated by adding 20 ml of ammonium hydroxide (28% w/w). The resulting polymer was precipitated in diluted HCl (pH 5 to 6, 40 °C) under high-dilution conditions, yielding a white precipitate that was collected by vacuum filtration. Residual low-molecular-weight impurities were removed through 72 h of dialysis against DI water, followed by lyophilization to obtain PVA-C5-DS.

### Preparation of IPS ink

Prior to ink formulation, the PVA-C5-DS precursor underwent a drying process in a vacuum oven maintained at 40 °C for a duration of 24 h. Subsequently, the ink was prepared at various concentrations (50 to 250 mg/ml) by combining the dried precursor with anhydrous DMSO under continuous magnetic agitation at room temperature. Following 24 h of dissolution, homogeneous colloidal systems exhibiting optical clarity to slight turbidity, which are IPS 3DP inks, were obtained. The PVA-C5-0.3 ink (200 mg/ml) served as the base matrix for preparing doped IPS 3DP inks through modification. Copper powder, CNTs, and HA were incorporated into the base ink at concentrations ranging from 20 to 400, 20 to 80, and 20 to 200 mg/ml, respectively. Following homogenization, the powder–matrix mixture underwent centrifugal mixing (5 min) to achieve uniform dispersion, yielding 3 modified ink formulations designated Cu-IPS, CNT-IPS, and HA-IPS inks. All modified inks required immediate use after preparation and were loaded into disposable syringes equipped with 150-μm-inner-diameter stainless steel needles. Extrusion filtration through these needles effectively removed larger particulate components prior to printing.

### IPS 3DP method

IPS 3DP was carried out via the Bio-Architect SR 3D manufacturing system (Regenovo Biotechnology Co., Ltd., Hangzhou, China). To ensure the optimal printability of the high-viscosity IPS inks, all formulations underwent a 6- to 12-h static degassing protocol prior to deposition to eliminate entrapped air bubbles. The homogenized bubble-free ink was subsequently loaded into 5-ml pneumatic cartridges equipped with conical plastic nozzles (210-μm inner diameter), where precise extrusion control (0.001-MPa resolution) was achieved through a digitally regulated air compressor system. The applied extrusion pressure was systematically varied between 0.01 and 0.2 MPa according to the specific rheological properties of each ink formulation. Owing to the deposition fidelity, the printing stage underwent meticulous levelling calibration, and disposable polystyrene Petri dishes serving as coagulation baths were horizontally secured on the platform to facilitate direct adhesion of 3D-printed scaffolds to the substrate. Postprint processing involved a sequential protocol: the scaffolds were first subjected to immersion in DI water for 6 h under ambient conditions with periodic solvent exchange (≥3 cycles) to completely leach residual DMSO, followed by delicate detachment from the substrate via tweezers. The harvested scaffolds were finally stored in a phosphate-buffered saline (PBS) solution at 4 °C to maintain structural integrity and biological compatibility until subsequent experimental utilization.

### Slow solvent exchange molding of PVA-C5 hydrogel

A DMSO solution of PVA-C5 for slow solvent exchange molding was prepared using the same method as that for the IPS ink, with a concentration of 50 mg/ml. The solution was slowly poured into a PTFE mold, ensuring that the liquid level height did not exceed 2 mm. The entire mold was placed into a storage box filled with water at the bottom, sealed, and kept at 25 °C. It is noted that the mold was positioned such that its height remained above the water level. Solvent exchange was induced by saturated water vapor in the sealed environment to trigger phase separation and molding. Depending on the amount of solution added, the process was sustained for 1 to 3 d, after which a preliminarily cross-linked PVA-C5 hydrogel was obtained. The hydrogel was then removed and immersed in abundant DI water to complete the solvent exchange thoroughly, yielding the PVA-C5 hydrogel.

### Surface and cross-sectional morphology of the IPS 3DP scaffolds

Following IPS 3DP completion, all samples were immersed in DI water for 24 h to extract soluble components. Cryogenic fracture preparation was subsequently conducted by quenching the hydrated scaffolds in liquid nitrogen until they reached thermal equilibrium, followed by manual fracturing via precooled forceps. The fractured samples were lyophilized in a freeze dryer at −80 °C for 48 h under 0.01 mbar vacuum. For SEM analysis, freeze-dried scaffolds were secured onto aluminum stubs via conductive carbon tape. A 5-nm platinum layer was deposited for 60 s to increase the surface conductivity. Microstructural evaluation of both the intact surfaces and fracture morphologies was performed via field-emission SEM (Gemini SEM 500, ZEISS, Germany) operated at an accelerating voltage of 5 kV and a working distance of 8 mm. All samples were fluorescently labeled with 5-(4,6-dichlorotriazinyl) aminofluorescein through covalent conjugation under dark conditions, following the standardized protocol established in our prior research.[9] High-resolution z-stack imaging was performed via a confocal laser scanning microscope (LSM 980, ZEISS, Germany). Consistent methodological parameters were used for PVA-C5 hydrogel characterization to ensure cross-sample comparability. Three-dimensional reconstructions were generated via the ZEN 3.2 software with optimized surface rendering algorithms.

### Modification of the scaffolds

Different modifications of the IPS 3DP scaffolds were achieved by soaking them in various solutions immediately after printing. Upon completion of the printing process, the coagulation bath was promptly removed, and the scaffolds were placed in Petri dishes containing 15 ml of DI water or 20%, 40%, or 60% (v/v) alcohol solutions. After soaking for 12 h, the scaffolds were carefully removed, rinsed under running water for 3 min, and then gently transferred with tweezers into fresh DI water for an additional 24 h of immersion.

### Mechanical performance testing

All IPS 3DP scaffold tensile samples were fabricated via a 3D bioprinter. The bioink at a concentration of 200 mg/ml was extruded through a 22G conical nozzle (inner diameter: 410 μm) to construct specimens measuring 40 mm in length, 10 mm in width, and 2 mm in thickness. The printing pattern consisted of 10 parallel filaments per layer along the longitudinal axis, with 5 stacked layers in total. Following printing, the scaffolds were immersed in DI water for 24 h to eliminate soluble impurities prior to subsequent characterization. For comparative analysis, PVA-C5 hydrogel control samples were fabricated via PTFE molds. Hydrogels with a 50 mg/ml polymer concentration were prepared as 4 cm × 4 cm square sheets, which were then sectioned into 4 cm × 1 cm rectangular strips for uniaxial tensile testing (Instron 5967, Instron Corporation, USA). Mechanical properties, including elongation at break and break strength, were determined through analysis of stress–strain curves, with triplicate measurements (*n* = 3) performed for each sample type.

### Electrical characterization

All IPS 3DP scaffolds were fabricated via a 3D bioprinter with 200 mg/ml bioink. The specimens were architecturally designed with nominal dimensions of 40 mm (length) × 10 mm (width) × 2 mm (thickness), employing a 22G conical extrusion nozzle (inner diameter: 410 μm). Postprinting protocols included 24 h of immersion in DI water to remove residual solutes, followed by vacuum drying until mass stabilization. Electrochemical characterization was performed in a Squidstat Plus workstation (Admiral Instruments, USA) under linear sweep voltammetry mode. A controlled potential of 1 V was applied during the measurements, with simultaneous recording of chronoamperometric curves capturing time–voltage–current triaxial responses. Triplicate tests (*n* = 3) were conducted for each scaffold configuration.

### Rheological tests

The rheological properties of the IPS 3DP ink were evaluated via an MCR-302 rotational rheometer (Anton Paar GmbH, Austria). The sample was clamped between 25-mm parallel plates with a gap distance of 400 μm. Viscosity measurements were conducted at a constant temperature of 25 °C under various shear rates (10 to 1,000 s^−1^).

### IPS 3DP scaffold recycling and ink regeneration

To achieve ink regeneration and recycling of IPS 3DP, the scaffolds were initially freeze-dried at −80 °C for 2 to 3 d to remove water and residual DMSO. The dried scaffolds were subsequently weighed and mixed with anhydrous DMSO at a predetermined mass ratio. After 24 h of dissolution at ambient temperature, regenerated IPS 3DP ink was obtained for subsequent 3D printing operations. This complete process from initial printing to ink regeneration was defined as one recycling cycle. Modified IPS 3DP inks underwent identical printing–recycling procedures via the same methodology. Notably, for regenerated modified IPS 3DP inks, inorganic components introduced during modification could be removed through additional centrifugation and filtration processes, yielding regenerated unmodified ink. The entire recycling protocol enabled repeated utilization of both pristine and modified IPS 3DP materials while maintaining printability.

### In vitro cell experiments

Cytotoxicity and scaffold biosafety assessments were conducted by coculturing rat BMSCs with 3D-printed scaffold extracts for 1, 3, and 5 d, following established protocols from previous literature [9]. For the cell seeding experiments, L-929 cells at passages 6 and 7 were suspended in complete medium to prepare a cell suspension at a density of 10,000 cells/ml. The sterilized scaffold samples and control samples were placed in 24-well plates, with 1 ml of cell suspension added to each well. After 48 h of incubation at 37 °C in a 5% CO_2_ atmosphere, the culture medium was removed, and the samples were subjected to vital staining with calcein AM/propidium iodide fluorescent dyes. The cell distribution patterns on and around the scaffolds were subsequently captured via an inverted fluorescence microscope.

### In vitro scaffold degradation

Following IPS 3DP completion, all scaffold samples were subjected to solvent purification through 48 h of immersion in excess DI water at ambient temperature with 6-h interval medium replacement. Vacuum drying was subsequently performed at 60 °C until constant mass attainment, with initial dry weights (*W*_0_) recorded via an analytical balance (±0.1 mg). The in vitro degradation study was conducted under physiological simulation conditions using enzyme-supplemented PBS (pH 7.4) at a 1:50 mass-to-volume ratio (1 g scaffold:50 ml medium). Each degradation system contained 2 U/ml collagenase type I and 100 U/ml hyaluronidase to mimic the enzymatic microenvironment. The samples were incubated in orbital shakers maintained at 37 °C. At 15-d intervals, triplicate samples were retrieved, gently rinsed with DI water, and vacuum-dried to a constant mass (*W_t_*) for quantitative mass loss percentage calculation [(*W*_0_ − *W_t_*)/*W*_0_ × 100%]. Fresh enzyme-buffered saline (PBS) was added after each sampling to maintain enzymatic activity. Following 90 d of incubation, macrostructural changes were documented through optical imaging. Mechanical integrity assessment was performed through uniaxial tensile testing according to a previously established protocol, with a minimum of triplicate samples per group (*n* ≥ 3).

### Statistical analysis

All experiments were replicated at least 3 times. Statistics are presented as mean ± standard deviation (*n* ≥ 3) and were analyzed via the GraphPad Prism 8 software. **P < 0.01 and ***P < 0.001.

## Data Availability

All data generated and analyzed in this study are included in the article.

## References

[1] Luo J, Li S, Xu J, Chai M, Gao L, Yang C, Shi X. Biomimetic strain-stiffening hydrogel with crimped structure. Adv Funct Mater. 2021;31(43): Article 2104139.

[2] Holzapfel GA, Humphrey JD, Ogden RW. Biomechanics of soft biological tissues and organs, mechanobiology, homeostasis and modelling. J R Soc Interface. 2025;22(222): Article 20240361. 39876788 10.1098/rsif.2024.0361 PMC11775666

[3] Eskandari F, Shafieian M, Aghdam MM, Laksari K. Tension strain-softening and compression strain-stiffening behavior of brain white matter. Ann Biomed Eng. 2021;49(1):276–286. 32494967 10.1007/s10439-020-02541-w

[4] Yuan C, Lü X, Bao W. Thermal protection performance of biomimetic flexible skin for deformable high-speed vehicles (DHSV-bio-FS) under uniaxial tensile strain. Research. 2025;7: Article 0394. 10.34133/research.0394 PMC11152053 38840900

[5] Yan B, Huang J, Han L, Gong L, Li L, Israelachvili JN, Zeng H. Duplicating dynamic strain-stiffening behavior and nanomechanics of biological tissues in a synthetic self-healing flexible network hydrogel. ACS Nano. 2017;11(11):11074–11081. 28956900 10.1021/acsnano.7b05109

[6] Prince E. Designing biomimetic strain-stiffening into synthetic hydrogels. Biomacromolecules. 2024;25(10):6283–6295. 39356204 10.1021/acs.biomac.4c00756

[7] Shao C, Meng L, Wang M, Cui C, Wang B, Han CR, Xu F, Yang J. Mimicking dynamic adhesiveness and strain-stiffening behavior of biological tissues in tough and self-healable cellulose nanocomposite hydrogels. ACS Appl Mater Interfaces. 2019;11(6):5885–5895. 30652853 10.1021/acsami.8b21588

[8] Zheng R, Zhong W, Chai M, Shi X. Dynamic compliance penis enlargement patch. Bioact Mater. 2024;42:194–206. 39285912 10.1016/j.bioactmat.2024.08.039 PMC11403245

[9] Chai M, Zhai Z, Liu X, Wu K, He Y, Ostrovidov S, Wu H, Bian L, Shi X. Bionic artificial penile *Tunica albuginea*. Matter. 2023;6(2):626–641.

[10] He C, He J, Wu C, Ruan C, Gu Q, Hao Y, Wu Y, Bai S, Han X, Ouyang L, et al. 3D printing for tissue/organ regeneration in China. Bio-Des Manuf. 2025;8:169–242.

[11] Wang S, Li S, Gao L. Dispersed association of single-component short-alkyl chains toward thermally programmable and malleable multiple-shape hydrogel. ACS Appl Mater Interfaces. 2019;11(46):43622–43630. 31674759 10.1021/acsami.9b16205

[12] Wang S, Liu M, Gao L, Guo G, Huo Y. Optimized association of short alkyl side chains enables stiff, self-recoverable, and durable shape-memory hydrogel. ACS Appl Mater Interfaces. 2019;11(21):19554–19564. 31062959 10.1021/acsami.9b06716

[13] Zhao L, Wang S, Yang Z, Tian L, Gao L, Shi X. Structural evolution of dispersed hydrophobic association in a hydrogel analyzed by the tensile behavior. Soft Matter. 2020;16(35):8245–8253. 32803214 10.1039/d0sm01211d

[14] Pan J, Gao L, Sun W, Wang S, Shi X. Length effects of short alkyl side chains on phase-separated structure and dynamics of hydrophobic association hydrogels. Macromolecules. 2021;54(13):5962–5973.

[15] Pan J, Zhang W, Zhu J, Tan J, Huang Y, Mo K, Tong Y, Xie Z, Ke Y, Zheng H, et al. Arrested phase separation enables high-performance keratoprostheses. Adv Mater. 2023;35(16): Article e2207750. 36680510 10.1002/adma.202207750

[16] Pan J, Zeng H, Gao L, Zhang Q, Luo H, Shi X, Zhang H. Hierarchical multiscale hydrogels with identical compositions yet disparate properties via tunable phase separation. Adv Funct Mater. 2022;32(13): Article 2110277.

[17] Peng T, Chai M, Chen Z, Wu M, Li X, Han F, Chen S, Liao C, Yue M, Song YQ, et al. Exosomes from hypoxia preconditioned muscle-derived stem cells enhance cell-free corpus cavernosa angiogenesis and reproductive function recovery. Adv Healthc Mater. 2024;13(28): Article e2401406. 39007245 10.1002/adhm.202401406

[18] Ye T, Chai M, Wang Z, Shao T, Liu J, Shi X. 3D-printed hydrogels with engineered nanocrystalline domains as functional vascular constructs. ACS Nano. 2024;18(37):25765–25777. 39231281 10.1021/acsnano.4c08359

[19] Liu G, Xia P, Kong W, Qiao T, Sun Y, Ren W, He Y. 3D printing of hard/soft switchable hydrogels. Int J Extrem Manuf. 2025;7(4): Article 045001.

[20] Wang Z, Zhang B, He Q, Chen H, Wang J, Yao Y, Zhou N, Cui W. Multimaterial embedded 3D printing of composite reinforced soft actuators. Research. 2025;6:0122. 10.34133/research.0122 PMC10202188 37223483

[21] Yao Y, Hui Y, Wang Z, Chen H, Zhu H, Zhou N. Granular ionogel particle inks for 3D printed tough and stretchable ionotronics. Research. 2025;6:0104. 10.34133/research.0104 PMC10246561 37292516

[22] Chai M, Zhong W, Yan S, Ye T, Zheng R, Yang Z, Shi X. Diffusion-induced phase separation 3D printed scaffolds for dynamic tissue repair. BMEMat. 2024;2(3): Article e12108.

[23] Karyappa R, Ohno A, Hashimoto M. Immersion precipitation 3D printing (*ip*3DP). Mater Horiz. 2019;6(9):1834–1844.

[24] Deore B, Sampson KL, Lacelle T, Kredentser N, Lefebvre J, Young LS, Hyland J, Amaya RE, Tanha J, Malenfant PRL, et al. Direct printing of functional 3D objects using polymerization-induced phase separation. Nat Commun. 2021;12(1): Article 55. 33397901 10.1038/s41467-020-20256-3 PMC7782741

[25] Dong Z, Cui H, Zhang H, Wang F, Zhan X, Mayer F, Nestler B, Wegener M, Levkin PA. 3D printing of inherently nanoporous polymers via polymerization-induced phase separation. Nat Commun. 2021;12(1): Article 247. 33431911 10.1038/s41467-020-20498-1 PMC7801408

[26] Sole-Gras M, Ren B, Ryder BJ, Ge J, Huang J, Chai W, Yin J, Fuchs GE, Wang G, Jiang X, et al. Vapor-induced phase-separation-enabled versatile direct ink writing. Nat Commun. 2024;15: Article 3058. 38594271 10.1038/s41467-024-47452-9 PMC11003993

[27] Chen Z, Wang H, Cao Y, Chen Y, Akkus O, Liu H, Cao C. Bio-inspired anisotropic hydrogels and their applications in soft actuators and robots. Matter. 2023;6(11):3803–3837.

[28] Zhao J, Hu Y, Li H, Liu C, Nie Z, Chen Z, Ling Q, Li Z, Zhao P, Song B, et al. Liquid–liquid phase separation-mediated cellular-scale compartmentalization of hydrogel covalent cross-linking promotes microtubule-based mechanosensing. J Am Chem Soc. 2025;147(17):14336–14347. 40252026 10.1021/jacs.5c00079

[29] Guo Y, Han Y, Cao Y, Chen Y, Xie J, Ding H, Liang S, Liu X, Sun W, Tang J, et al. Facile fabrication of tough super macroporous hydrogel via enhanced phase separation. Adv Funct Mater. 2025;35(2): Article 2412412.

[30] Zhang D, Tang Y, Zhang K, Xue Y, Zheng SY, Wu B, Zheng J. Multiscale bilayer hydrogels enabled by macrophase separation. Matter. 2023;6(5):1484–1502.

[31] Shi W, Li Z, Peng L, Wang X, Zheng F, Su T, Huang Q, Cao L, Zheng A. Organic-inorganic nHA-gelatin/alginate high strength macroporous cryogel promotes bone regeneration. Smart Mater Med. 2024;5(3):337–347.

[32] Dargusch MS, Yang N, Balasubramani N, Venezuela J, Liu S, Jing L, Sen Y, Qu J, Wang G, Cairney J. Magnesium-based bioceramic-enhanced composites fabricated via friction stir processing. Smart Mater Med. 2024;5(3):447–459.

[33] Mojumder MRH, Kim S, Yu C. Soft artificial synapse electronics. Research. 2025;8:0582. 39877465 10.34133/research.0582 PMC11772661

[34] Lv C, Zhou Z, Li Y, Lu S, Bai Y. Multi-responsive shape memory porous composites for self-powered sensors and self-sensing actuators. Chem Eng J. 2023;477: Article 147059.

[35] Huang S, Xiao R, Lin S, Wu Z, Lin C, Jang G, Hong E, Gupta S, Lu F, Chen B, et al. Anisotropic hydrogel microelectrodes for intraspinal neural recordings in vivo. Nat Commun. 2025;16(1): Article 1127. 39875371 10.1038/s41467-025-56450-4 PMC11775234

[36] Zhao Z, Ji J, Zhang Y, Liu J, Yu R, Yang X, Zhao X, Huang W, Zhao W. Ultra-elastic conductive silicone rubber composite foams for durable piezoresistive sensors via direct ink writing three-dimensional printing. Chem Eng J. 2025;504: Article 158733.

[37] Yu H, Bian J, Chen F, Li K, Huang Y. Laser-guided, self-confined graphitization for high-conductivity embedded electronics. Research. 2025;7: Article 0305. 10.34133/research.0305 PMC11020139 38628354

[38] He C, Qiao T, Ren X, Xie M, Gao Q, Xie C, Wang P, Sun Y, Yang H, He Y. Printability in multi-material projection-based 3-dimensional bioprinting. Research. 2025;8: Article 0613. 40041038 10.34133/research.0613 PMC11876545

[39] Jiang M, Zheng J, Tang Y, Liu H, Yao Y, Zhou J, Lin W, Ma Y, Liu J, Zhou J. Retrievable hydrogel networks with confined microalgae for efficient antibiotic degradation and enhanced stress tolerance. Nat Commun. 2025;16: Article 3160. 40175365 10.1038/s41467-025-58415-z PMC11965497

[40] Zhou L, Forman HJ, Ge Y, Lunec J. Multi-walled carbon nanotubes: A cytotoxicity study in relation to functionalization, dose and dispersion. Toxicol In Vitro. 2017;42:292–298. 28483489 10.1016/j.tiv.2017.04.027 PMC5577988

