# Supplementary material for: Immersion Phase Separation 3-Dimensional Printing for Strain-Stiffening Hydrogel Scaffolds
Source: Research (Wash D C). 2025 Jun 17;2025:0742. doi: 10.34133/research.0742 (PMC12172163; doi:10.34133/research.0742)
Supplement: Supplementary 1 — Figs. S1 to S10 Movies S1 to S3 [file research.0742.f1.zip › sm.docx]

**Supplemental Figures**

**
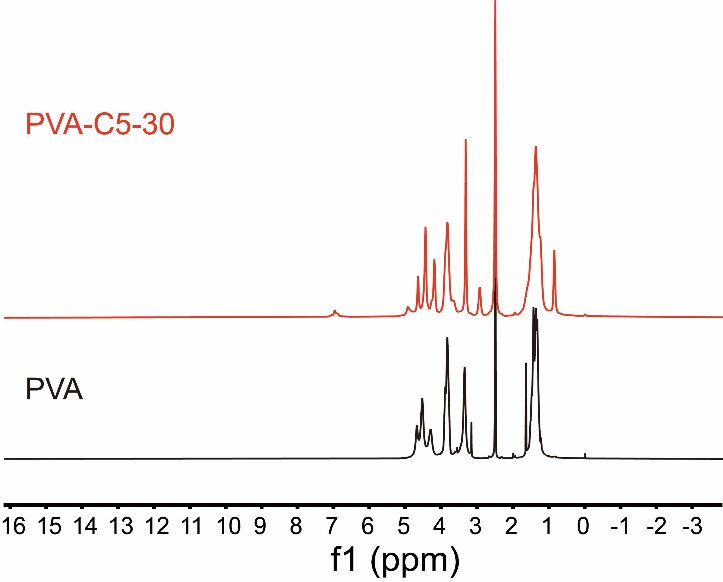
**

**Fig. S1.** H^1^ NMR spectra of PVA and PVA-C5-30.

**
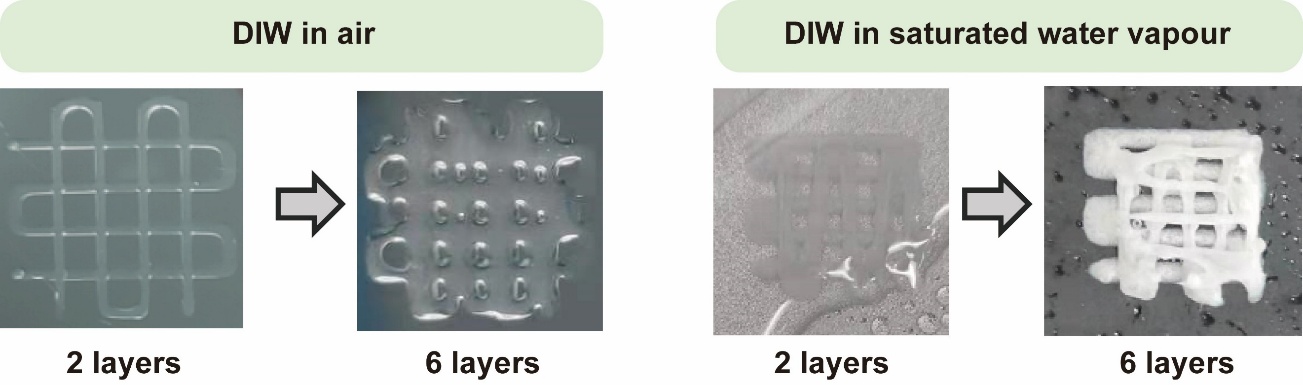
**

**Fig. S2.** Morphological evolution of DIW-printed IPS 3DP scaffolds (stage-specific structural features under ambient air vs. saturated water vapor environments).


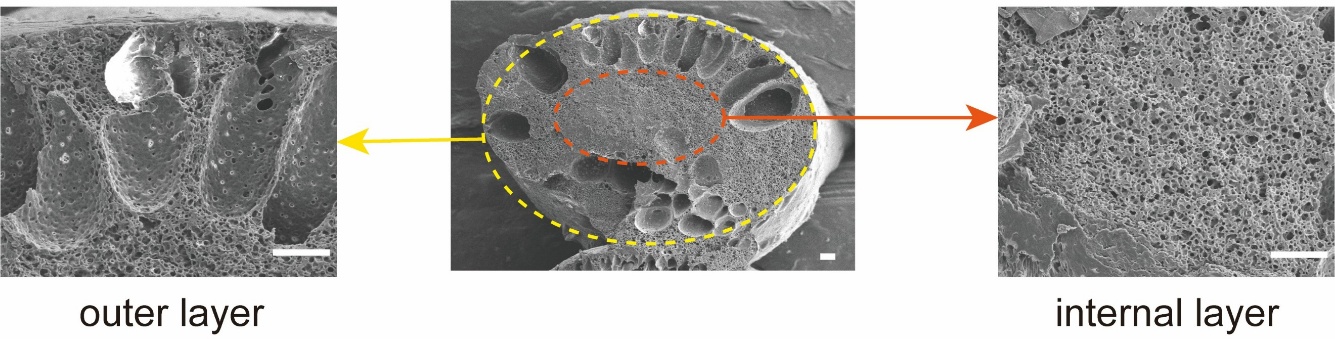


**Fig. S3.** Representative SEM images of IPS 3DP scaffolds demonstrating the cross-sectional morphology of individual filaments, with distinct core-shell architectural features (scale bar: 20 μm).


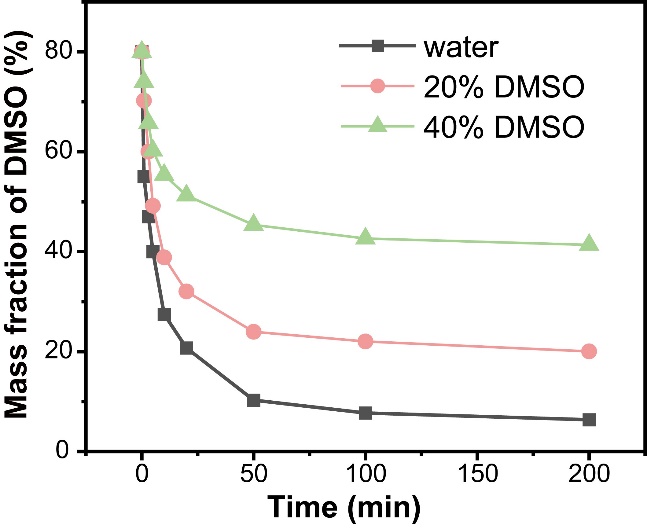


**Fig. S4.** At room temperature, 0.1 mL of IPS 3DP ink (200 mg/mL) was extruded and immersed in 20 mL coagulation baths containing deionized water, 20% DMSO solution, or 40% DMSO solution to monitor temporal changes in DMSO mass fraction within the ink/hydrogel system (n = 3).

**
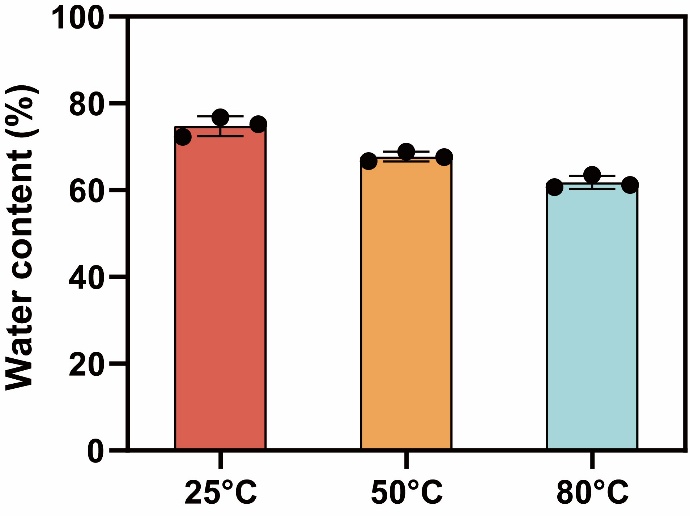
**

**Fig. S5.** Water content of IPS 3DP scaffolds subjected to thermal treatment at varying temperatures (25°C, 50°C, 80°C) following fabrication under identical conditions.

**
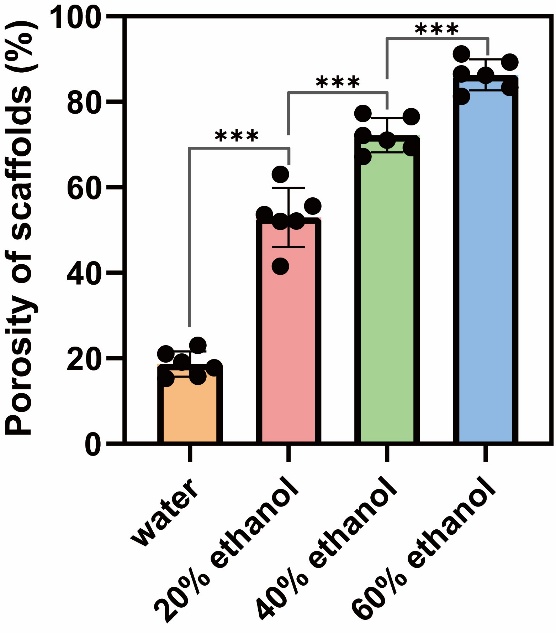
**

**Fig. S6.** Porosity of IPS 3DP scaffolds post-treated with varying composition coagulation baths (deionized water and 20%, 40%, 60% ethanol solutions) (***p < 0.001).


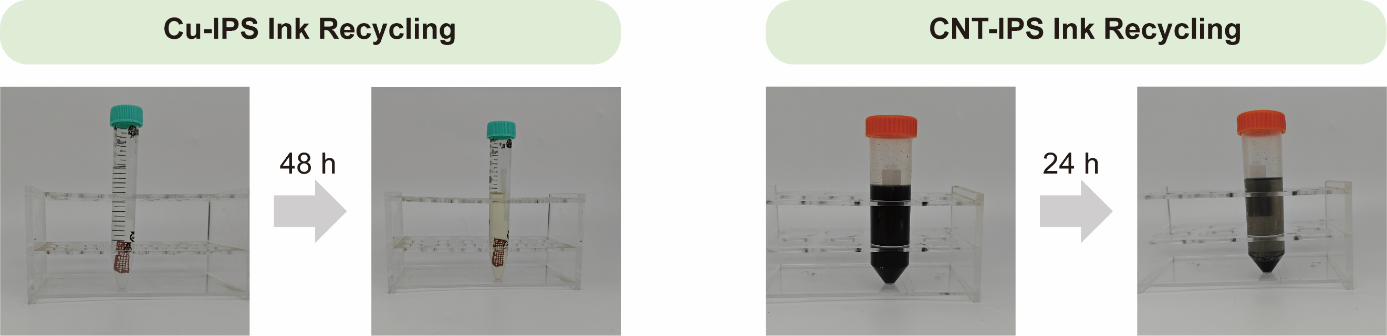


**Fig. S7.** Cu-IPS inks and CNT-IPS inks at different stages of the recycling and reclamation process.


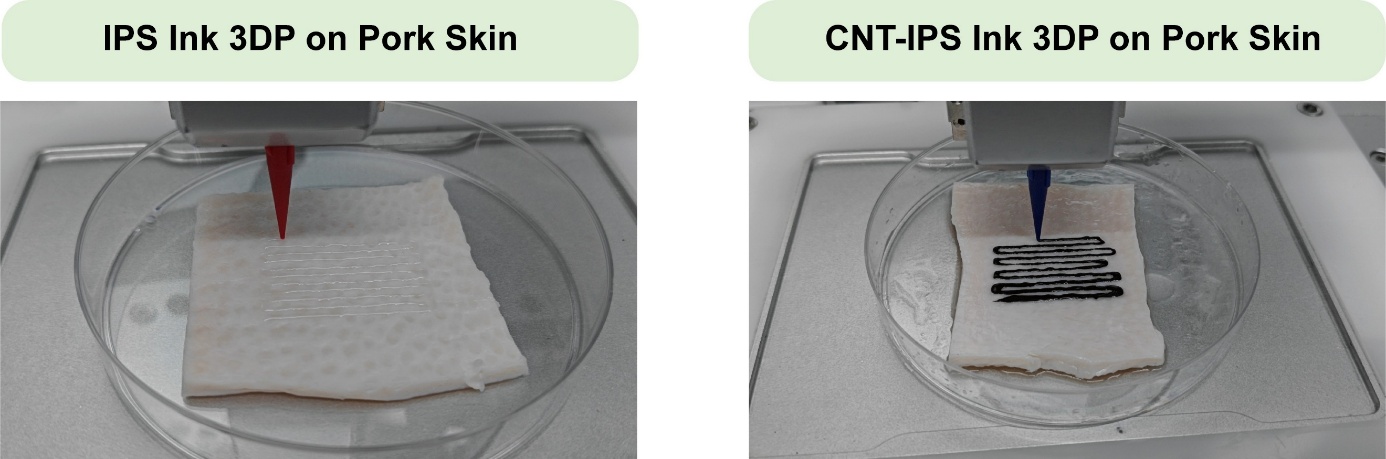


**Fig. S8.** IPS inks and CNT-IPS inks were used for DIW 3D printing directly on moist pigskin.


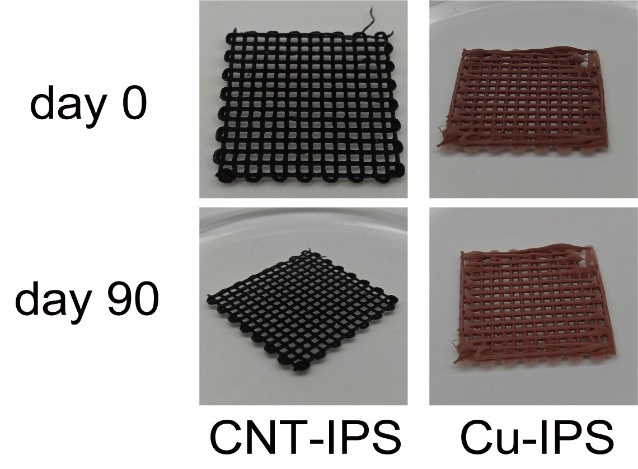


**Fig. S9.** Macroscopic photographs of CNT-IPS scaffold and Cu-IPS scaffold before and after 90 days of *in vitro* degradation.


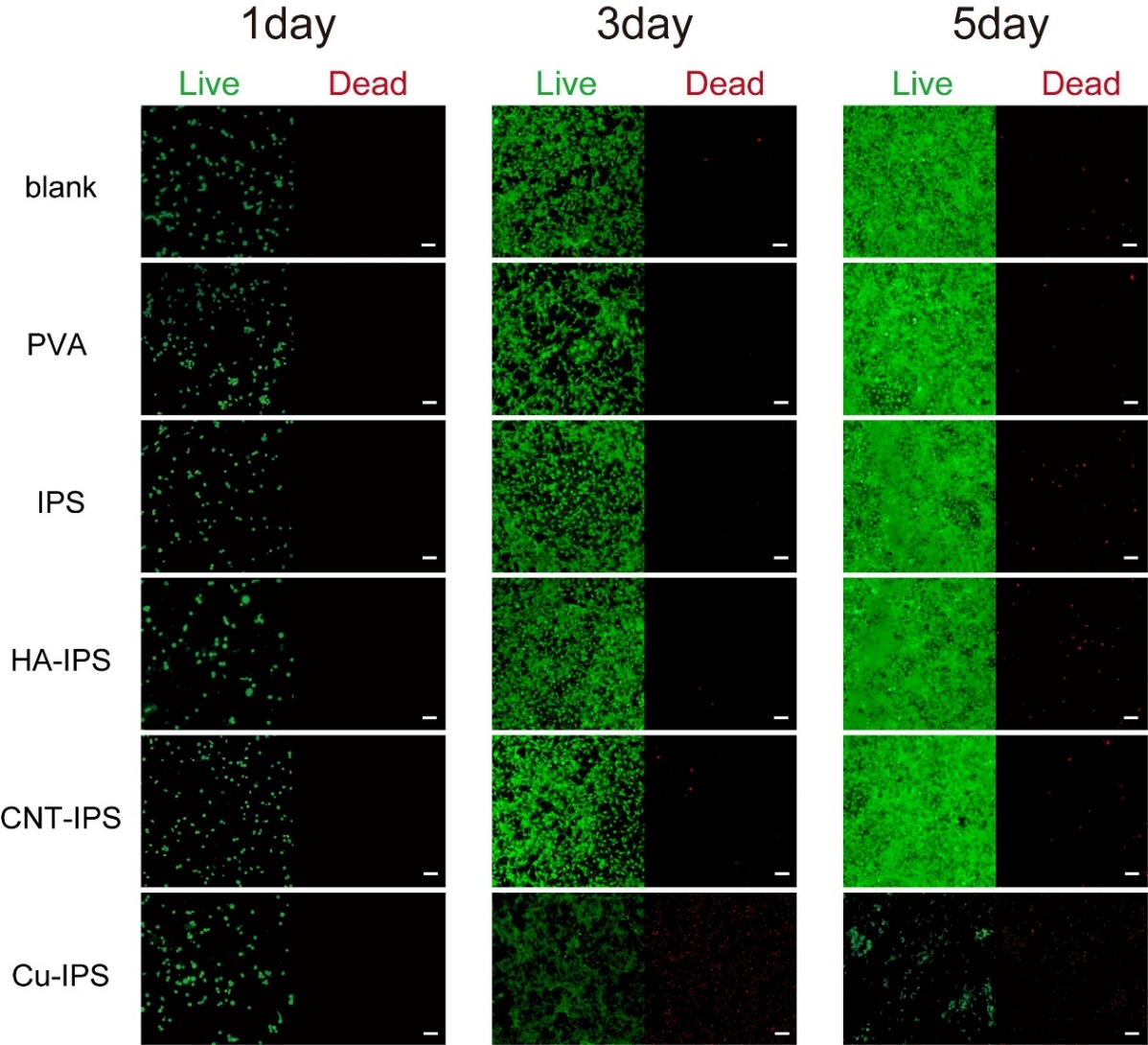


**Fig. S10.** Live/Dead fluorescence images of BMSCs cultured in scaffold-conditioned media for 1, 3, and 5 days (Scale bar: 50 μm).

**Movie S1. Comparison of DIW printing with IPS inks in air and water bath.**

**Movie S2. The phenomenon of solvent exchange in IPS 3DP inks in coagulation baths of different compositions.**

**Movie S3. IPS 3D printing process of inorganic filler modified inks.**
